# *TilGAN*: GAN for Facilitating Tumor-Infiltrating Lymphocyte Pathology Image Synthesis With Improved Image Classification

**DOI:** 10.1109/access.2021.3084597

**Published:** 2021-05-28

**Authors:** MONJOY SAHA, XIAOYUAN GUO, ASHISH SHARMA

**Affiliations:** 1 Department of Biomedical Informatics, School of Medicine, Emory University, Atlanta, GA 30322, USA; 2 Department of Computer Science, Emory University, Atlanta, GA 30332, USA

**Keywords:** Digital pathology, deep learning, generative adversarial network, lung cancer, artificial intelligence

## Abstract

Tumor-infiltrating lymphocytes (TILs) act as immune cells against cancer tissues. The manual assessment of TILs is usually erroneous, tedious, costly and subject to inter- and intraobserver variability. Machine learning approaches can solve these issues, but they require a large amount of labeled data for model training, which is expensive and not readily available. In this study, we present an efficient generative adversarial network, *TilGAN*, to generate high-quality synthetic pathology images followed by classification of TIL and non-TIL regions. Our proposed architecture is constructed with a generator network and a discriminator network. The novelty exists in the *TilGAN* architecture, loss functions, and evaluation techniques. Our *TilGAN*-generated images achieved a higher Inception score than the real images (2.90 vs. 2.32, respectively). They also achieved a lower kernel Inception distance (1.44) and a lower Fréchet Inception distance (0.312). It also passed the Turing test performed by experienced pathologists and clinicians. We further extended our evaluation studies and used almost one million synthetic data, generated by *TilGAN*, to train a classification model. Our proposed classification model achieved a 97.83% accuracy, a 97.37% F1-score, and a 97% area under the curve. Our extensive experiments and superior outcomes show the efficiency and effectiveness of our proposed *TilGAN* architecture. This architecture can also be used for other types of images for image synthesis.

## I. INTRODUCTION

Tumor infiltrating lymphocytes (TILs) play a significant role in cancer diagnosis and prognosis [1]. The presence of TILs in different cancer types (such as lung, colon, and breast cancer) signifies improved clinical outcomes and faster response to chemotherapy [2]. Recent evidence has emerged that the infiltration of antitumor type I lymphocytes can improve cancer prognosis [3]. TILs are a special white blood cell that shows a tendency to emigrate towards tumor cells from the bloodstream [4]. TILs comprise mainly T cells, B cells, mononuclear cells, and polymorphonuclear immune cells (such as neutrophils, eosinophils, and basophils) [5]. TILs normally float around tumor cells.

**FIGURE 1.**
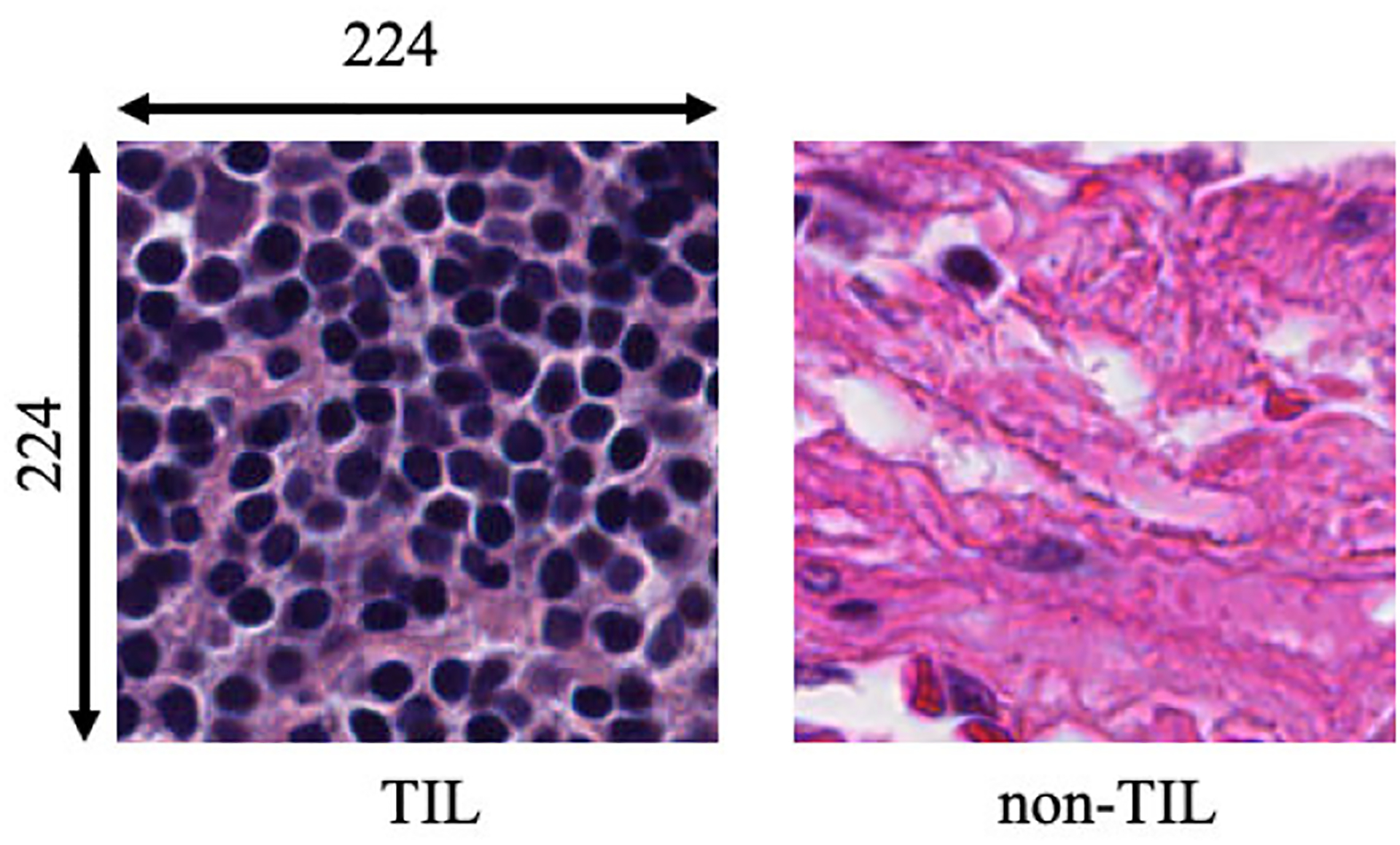
Original tumor-infiltrating lymphocyte (TIL) and non-tumor-infiltrating lymphocyte (non-TIL) patches.

As per the World Health Organization and American Cancer Society, lung cancer is one of the most devastating cancers globally, accounting for almost 14% of new cancers in men and 13% of new cancers in women in the United States [6]. It has also been reported that in 2019, lung cancer caused approximately 228,150 new cases (116,440 men and 111,710 women) and 142,670 deaths (76,650 men and 66,020 women) in the United States [7]. For lung cancer prognosis, pathological image analysis is considered the primary and gold standard screening method. For this purpose, pathologists collect a small part of the tissue from the suspected tumor region. Next, the tissues are further processed and stained using different stains, including hematoxylin and eosin (H&E) [8], [9]. Lung cancer pathology images typically contain TILs, tumor cells, mitotic cells, stroma, etc. Under a microscope, TILs appear with round, deep bluish nuclei [10]. The details of the TIL and non-TIL regions are shown in figure 1. Pathologists follow manual image analysis procedures to analyze the tissue regions. This procedure fully depends on the knowledge of the pathologists. Moreover, it is costly and time consuming.

Deep learning has shown promising results in image analysis, signal analysis, video analysis, and many more fields [9]. Currently, this method is one of the most popular machine learning approaches and is used to solve many complicated tasks, such as object classification, image segmentation, and risk prediction. However, it has a few disadvantages. The most significant one is that deep learning requires a large amount of data to meet satisfactory performance [11]. However, biomedical data are expensive and not readily available, as approval from the patients and institutional review board are required to use them. Biomedical data may also contain artifacts, noise, etc., which also reduce the total number of data points.

To solve the data availability problem, the authors of [12] proposed a generative adversarial network (GAN) for natural image synthesis. The GAN comprises mainly a generator network/model and a discriminator network/model. The generator network generates synthetic/fake data, which looks like real data, while the discriminator checks the quality of the synthetic data. Categorically, the generator network learns from latent space, and the discriminator differentiates the real and synthetic data distributions [13]. The generator attempts to fool the discriminator by increasing the generator loss [14]. In this study, we present an efficient generative adversarial network, *TilGAN*, to generate high-quality synthetic pathology data of TIL and non-TIL regions to mitigate data imbalances, to improve the classification accuracy, and finally to assist pathologists and clinicians in their decision-making processes. The main novelty exists in *TilGAN* architecture, loss functions, and evaluation techniques.

This manuscript has five sections. Section I introduces the work, and Section II discusses related work. In Section III, the materials and methods are discussed. Sections IV and V discuss the results and present the discussion and conclusion, respectively.

## II. RELATED WORK

A GAN, an unsupervised method, is used to generate millions of synthetic data, which resembles the real dataset [15]. Traditional generative models follow the rules of explicit approximation inference and Markov fields, but GANs do not follow this rule. The generative network of GAN produces high-quality fake data to mislead the discriminator. The training process of the GAN ends when a Nash equilibrium from game theory is reached [16]. Hence, the GAN learning process is considered a minimum-maximum optimization problem.

Initially, a GAN was developed for natural image synthesis [12], but gradually, the default architecture was changed to improve the synthetic image quality and to solve other data processing issues, including color enhancement [34], image translation [35], [36], nuclei segmentation [37], [38], cell-level visual representation [39], and image classification [40]. Various researchers have proposed different cost functions for the generator and discriminator networks to improve the quality of synthetic images, such as relativistic GAN, hinge GAN [41], relativistic average GAN [42], and Wasserstein GAN [43]. The main difference between the standard GAN and the modified GANs is that the standard GAN tries to prove that the input data are real, whereas modified GANs measure the probability that generated data are less realistic than the real data (or vice versa). With a standard GAN, the discriminator squeezes the output into two ends, i.e., 0 or 1. Modified GANs measure the distances or differences between fake and real images [44]. When the discriminator reaches an optimum level, gradients vanish. Many new GAN architectures have been proposed for natural and biomedical image synthesis. Cycle-consistency GANs are one of the most common GAN architectures and was designed for biomedical image synthesis [45], image-to-image translation [46], etc.

The authors of [47] proposed a GAN architecture for stain transfer or stain normalization. Their architecture was trained with a multiobjective cost function to learn image-specific color transformations and dataset-specific staining properties. StainGAN [34] and InfoGAN [48] were also used for color normalization on WSIs in different studies. Pathology GAN was proposed for pathology image synthesis [49] with a Fréchet Inception distance of 16.65. This architecture was developed using BigGAN as the baseline architecture [50]. A GAN was also applied to radiology image synthesis and translation. The authors of [51] suggested an edge-aware GAN [51] for MRI image synthesis. Task-driven GAN was proposed in [52] for X-ray image synthesis. The authors of [53] proposed a GAN for computed tomography (CT) to magnetic resonance image (MRI) data synthesis and translation. A conditional GAN was used for PET image synthesis [54]. A deep convolutional GAN (DCGAN) was recommended for image synthesis and the detection of liver cancer on X-ray and CT images [55]. Table 1 refers to the characterization of existing GAN architectures. Here, we have summarized a few GAN architectures that are used for image synthesis, image translation, color normalization, etc.

In this manuscript, we perform image synthesis with *TilGAN*, which is constructed using different baseline architectures, such as Pathology GAN [49], BigGAN [50], a cycle-consistency GAN [56], and a relativistic average GAN [42].

Pathology images show important information, and small changes in the tissue characteristics may result in a wrong diagnosis and patient death. Therefore, it is a very challenging task to maintain the real image characteristics of synthetic images. Existing GAN architectures generate TIL and non-TIL patches, but our proposed network shows improved results. We targeted preserving real image features such as image appearance, chromatin information, stain colors, and tissue contents.

In summary, the novel technical contributions of this study can be summarized as follows:

- The most important contribution of this study lies in the architecture of *TilGAN*. Due to its novel architecture, *TilGAN* generates millions of high-quality, clinically significant TIL patches.
- Second, we propose a modified version of the relativistic average cost function to preserve important pathological signatures.
- Third, to our knowledge, this is the first report to propose a GAN that specifically aims to generate TIL and non-TIL patches.
- Fourth, the generated synthetic images are used for classification model training.

The detailed method, along with the results, will be discussed in the subsequent sections.

## III. MATERIALS AND METHODS

### A. DATASET

**FIGURE 2.**
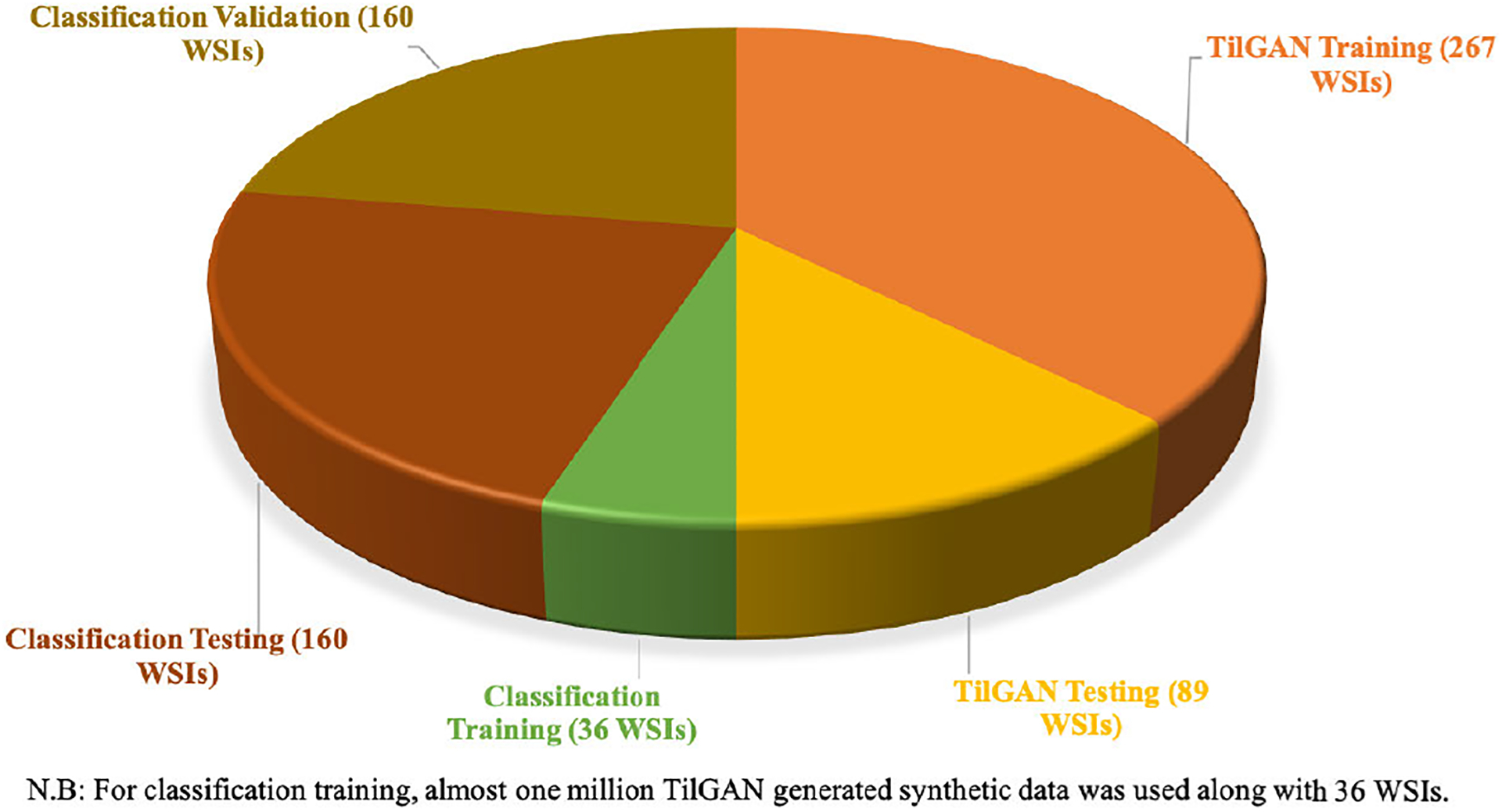
Whole slide images (WSIs) distribution chart.

In total, 712 H&E stained WSIs of lung cancer (356 adenocarcinomas and 356 squamous cell carcinomas) were collected from The Cancer Genome Atlas data repository (https://tcga-data.nci.nih.gov/tcga/). This is a public repository, and the data are freely available for research. For our study, the collected data were equally split into two sets, with zero overlaps. One half of the data, i.e., 356 WSIs (178 adenocarcinomas and 178 squamous cell carcinomas), was used for *TilGAN*, and the other half was used for classification purposes. Out of the 356 WSIs, we used 75% (267 WSIs) for training and 25% (89 WSIs) for testing of the *TilGAN* architecture. The ground truths were generated by experts using HistomicsTK (https://digitalslidearchive.github.io/HistomicsTK/). To train our classification model, we used one million high-quality synthetic images generated by *TilGAN* of size 224 × 224 pixels and 10% (i.e., 36 WSIs out of the remaining 356 WSIs) real labeled data. The rest of the 320 WSIs were split into testing (50%) and validation sets (50%) to evaluate the classification model. In the figure 2, the WSIs distribution chart has been shown.

### B. TilGAN METHODOLOGY

**FIGURE 3.**
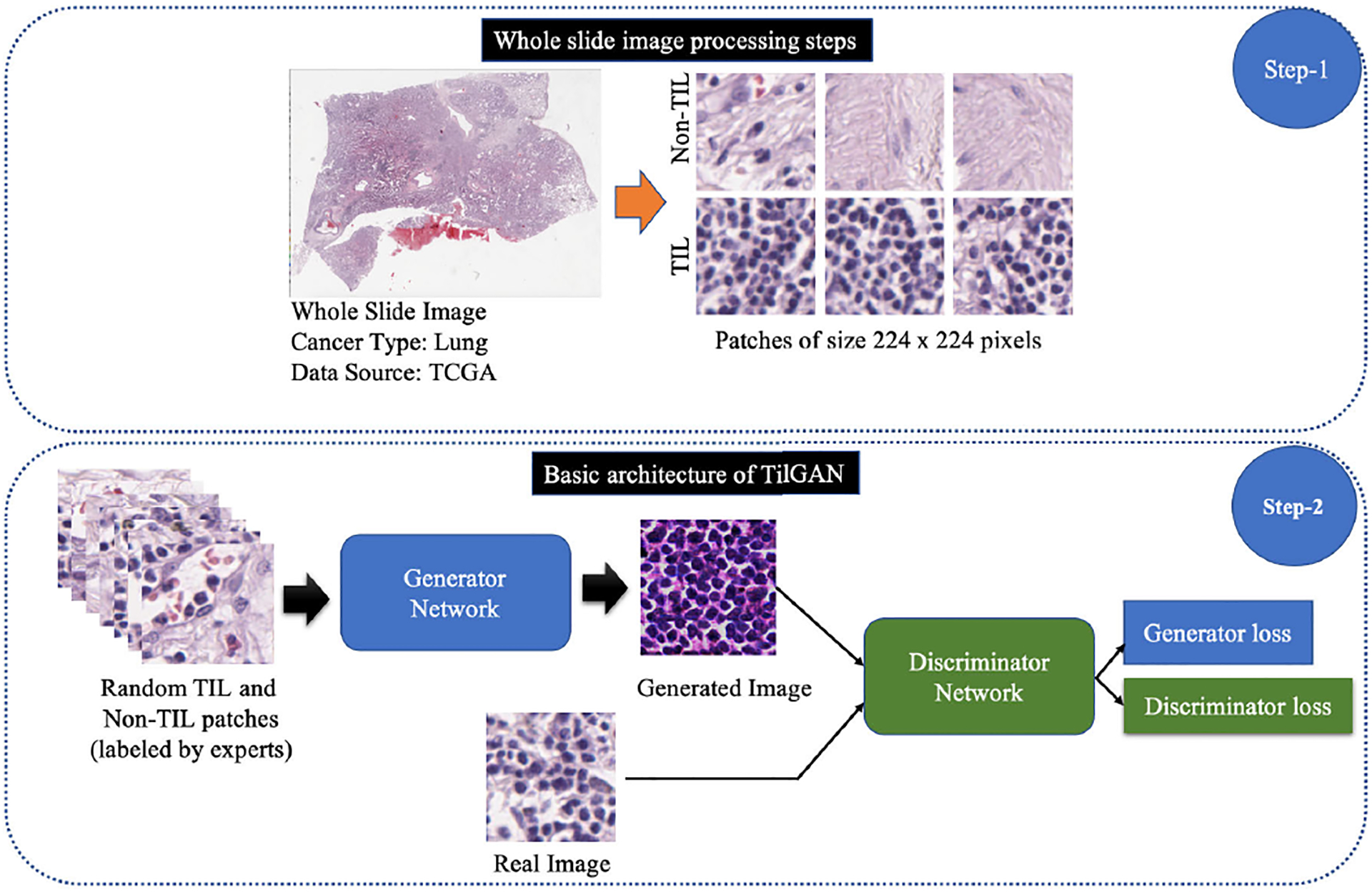
The workflow diagram of our proposed *TilGAN* architecture. At step 1, H&E-stained WSIs were processed to extract TIL and non-TIL patches of size 224 × 224 pixels. At step 2, the input of the generator network randomly selected real TIL and non-TIL patches, and the output of the generator was synthetic TIL and non-TIL patches of the same size. The inputs of the discriminator were real and synthetic TIL and non-TIL patches. The discriminator network was used to discriminate real and fake TIL and non-TIL cases. The training of the *TilGAN* model was performed twice, as there were two types of data: TIL and non-TIL data.

We developed the *TilGAN* architecture for the synthesis of TIL and non-TIL pathology images of size 224 × 224 pixels. This network was trained from scratch. We adopted a supervised learning strategy that uses hand-labeled images. There are many GAN architectures available for natural image synthesis, but few of them have been used for pathology image generation. Pathology images carry essential clinical features about cell nuclei, stroma, mitosis, lymphocytes, etc. Hence, image synthesis using pathology data requires special skills. Small changes in the visual appearance of nuclei, lymphocytes, etc., may change the clinical meaning. The workflow diagram of our proposed *TilGAN* architecture is shown in figure 3.

#### 1) TilGAN ARCHITECTURE DETAILS

The *TilGAN* architecture comprised a generator network *G*_*NET*_ and a discriminator network *D*_*NET*_. The input of the generator was randomly chosen from the annotated real TIL and non-TIL patches of size 224 × 224 pixels. The output of the generator was synthetic TIL and non-TIL patches. The generator was defined as a mapping function *z* to learn the generator’s distribution over the data *y*. The discriminator, *D*_*NET*_, showed the probability that *y* was more realistic than the generator’s distribution. The generator network of *TilGAN* comprised six convolutional layers, five up-convolution layers, and two dense layers. The up-convolution, is obtained by a transposed convolution, operation increased the height and width of the feature maps by two. The discriminator of *TilGAN* was formed using six convolutional layers, six down-convolution layers, and two dense layers. The down-convolution, general convolution, operation decreased the height and width of the feature maps by two. This design helped the model learn the features from real images efficiently and effectively. Different learning rates were used for the generator and discriminator networks. The overfitting issue was tracked by incorporating more data and varying the dropout layers. During training, we set the dropout value to 0.5. The rectified linear unit (ReLu) activation function was used after each convolution layer.

The detailed architecture of our proposed *TilGAN* model is shown in table 2.

#### 2) MODIFIED LOSS FUNCTION FOR THE *TilGAN* ARCHITECTURE

Pathology images possess distinct types of textural, color, and morphological features, which are linked with the patient’s diagnosis and prognosis. Hence, it is essential to handle these types of data separately, unlike other nonclinical data. The existing loss functions of GAN architectures generated TIL and non-TIL patches, but the clinical features of those images were not consistent. Hence, we developed a modified version of the relativistic average loss function to solve these issues. We used the modified relativistic average cost function for both networks (generator and discriminator). The fundamental theory of the modified relativistic average loss function originates from the binary cross-entropy loss function [12] as follows:

(1) $$\text{Loss}\left({\hat{O}}_{d},O_{d}\right)=O_{d}\cdot \text{log}\;{\hat{O}}_{d}+\left(1-O_{d}\right)\cdot \text{log}\left(1-{\hat{O}}_{d}\right)$$

Here, *O*_*d*_ and ${\hat{O}}_{d}$ denote the original and reconstructed data, respectively. When training the discriminator, the labeled data from the original data distribution *P*_*image*_(*y*) are *O*_*d*_ = 1 (when the data are real) and ${\hat{O}}_{d}=D_{NET}\left(y\right)$. Substituting these values into equation 1, we obtain

(2) $$\text{Loss}\left(D_{NET}\left(y\right),\;1\right)=1\;\cdot \;\text{log}\left(D_{NET}\left(y\right)\right)+(1-1)\;\cdot \;\text{log}\left(1-D_{NET}\left(y\right)\right)\;\Rightarrow \text{Loss}\left(D_{NET}\left(y\right),\;1\right)=\text{log}\left(D_{NET}\left(y\right)\right)$$

When the data are fake, the values of *O*_*d*_ and ${\hat{O}}_{d}$ will be 0 and *D*_*NET*_ (*G*_*NET*_ (*z*)), respectively. Substituting these values into equation 1, we obtain

(3) $$\text{Loss}\left(D_{NET}\left(G_{NET}\left(z\right)\right),\;0\right)=0\cdot \text{log}\;D_{NET}\left(G_{NET}\left(z\right)\right)+\left(1-0\right)\cdot \text{log}\;\times \;\left(1-D_{NET}\left(G_{NET}\left(z\right)\right)\right)\;\Rightarrow \text{Loss}\left(D_{NET}\left(G_{NET}\left(z\right)\right),0\right)=\text{log}\left(1-D_{NET}\right.\left.\left(G_{NET}\left(z\right)\right)\right)$$

The main purpose of the discriminator *D*_*NET*_ is to distinguish real and fake images. Hence, equations 2 and 3 should be maximized. Next, the discriminator loss will be as follows:

(4) $$Loss^{\left(D_{NET}\right)}=\text{max}\left[\text{log}\;D_{NET}\left(y\right)+\text{log}\left(1-D_{NET}\left(G_{NET}\left(z\right)\right)\right)\right]$$

The final generator *G*_*NET*_ loss will be

(5) $$Loss^{\left(G_{NET}\right)}=\text{min}\left[\text{log}\;D_{NET}\left(y\right)+\text{log}\left(1-D_{NET}\right.\right.\left.\left.\left(G_{NET}\left(z\right)\right)\right)\right]$$

If we combine equations 4 and 5, we obtain equation 6. However, equation 6 is valid only for a single data point.

(6) $$\text{Loss}\;=\underset{G_{NET}}{\text{min}}\;\underset{{}_{D_{NET}}}{\text{max}}\left[\text{log}D_{NET}\left(y\right)\right.\left.+\text{log}\left(1-D_{NET}\left(G_{NET}\left(z\right)\right)\right)\right]$$

To consider the entire large amount of data, equation 6 can be modified as below: [57]–[59],

(7) $$\underset{G_{NET}}{\text{min}}\;\underset{{}_{D_{NET}}}{\text{max}}\;V\left(G_{NET},\;D_{NET}\right)=E_{y\sim P_{\text{image}}\left(y\right)}\left[\text{log}\;D_{NET}\left(y\right)\right]+E_{z\sim P_{z}\left(z\right)}\left.\times \left[\text{log}\left(1-D_{NET}\left(G_{NET}\left(z\right)\right)\right)\right]\right)$$

The loss functions of a standard GAN can be classified into saturating and non-saturating loss functions [42]. Equation 7 is an example of a non-saturating loss function. In the case of saturating loss, the equation for the discriminator will be

(8) $$Loss^{\left(D_{NET}\right)}=-E_{y\sim P_{\text{image}}\left(y\right)}\left[\text{log}D_{NET}\left(y\right)\right]-E_{z\sim P_{z}\left(z\right)}\left[\text{log}\left(1-D_{NET}\left(G_{NET}\left(z\right)\right)\right)\right]$$

In the standard GAN, *D*_*NET*_ (*y*) has been represented as a *D*_*NET*_ (*y*) = *sigmoid*(*C*(*y*)) [60], [61]. Here, *C*(*y*) determines the possibility of having real or fake data. Hence, it is also known as a critic or non-transformed discriminator output. If the value of *C*(*y*) is negative, then the input data are fake, and vice versa. After substituting the value of *D*_*NET*_ (*y*) into equation 8, we obtain

(9) $$Loss^{\left(D_{NET}\right)}=-E_{y\sim P_{\text{image}}\left(y\right)}\left[\text{log}\left(\text{sigmoid}\right.\left(C\left(y\right)\right)\right]-E_{z\sim P_{z}\left(z\right)}\left[\text{log}\left(1-D_{NET}\left(G_{NET}\left(z\right)\right)\right)\right]$$

With a relativistic standard GAN, we compute the distance, which depends on the real and fake data distribution. Hence, *D*_*NET*_ (*y*) will change to *D*_*NET*_ (*y*_*r*_*, y*_*f*_) = *sigmoid*(*D*_*NET*_ (*y*_*r*_) − *D*_*NET*_ (*y*_*f*_). Here, *r* and *f* indicate real and fake data, respectively. Now, the discriminator loss will be:

(10) $$Loss^{\left(D_{NET}\right)}=-E_{\left(y_{r},\;y_{f}\right)\sim \left(\mathbb{R},\;\mathbb{N}\right)}\left[\;log\;\left(\;\text{sigmoid}\;\left(D_{NET}\left(y_{r}\right)\right.\right.\right.\left.\left.\left.-D_{NET}\left(y_{f}\right)\right)\right)\right]$$

and the generator loss will be:

(11) $$Loss^{\left(G_{NET}\right)}=-E_{\left(y_{r},\;y_{f}\right)\sim \left(\mathbb{R},\;\mathbb{N}\right)}\left[\;log\;\left(\;\text{sigmoid}\;\left(D_{NET}\left(y_{f}\right)\right.\right.\right.\left.\left.\left.-D_{NET}\left(y_{r}\right)\right)\right)\right]$$

Here, *D*_*NET*_ (*y*_*r*_) = *D*_*NET*_ (*y*_*f*_) = 0.5 has been set as an optimal point [61]. Equation 7 can also be generalized as

(12) $$Loss^{\left(D_{NET}\right)}=E_{y_{r}\sim \mathbb{R}}\left[f_{1}\left(D_{NET}\left(y_{r}\right)\right)\right]+E_{z\sim {\mathbb{R}}_{z}}\left.\left[f_{2}D_{NET}\left(G_{NET}\left(z\right)\right)\right)\right]$$

(13) $$Loss^{\left(G_{NET}\right)}=E_{y_{r}\sim \mathbb{R}}\left[g_{1}\left(D_{NET}\left(y_{r}\right)\right)\right]+E_{z\sim {\mathbb{R}}_{z}}\left.\left[g_{2}D_{NET}\left(G_{NET}\left(z\right)\right)\right)\right]$$

Here, functions *f* and *g* map a scalar input to another scalar. The corresponding relativistic cost function will be as follows:

(14) $$Loss^{\left(D_{NET}\right)}=E_{\left(y_{r},\;y_{f}\right)\sim \left(\mathbb{R},\;\mathbb{N}\right)}\left[f_{1}\left(D_{NET}\left(y_{r}\right)-D_{NET}\left(y_{f}\right)\right)\right]+E_{\left(y_{r},\;y_{f}\right)\sim \left(\mathbb{R},\;\mathbb{N}\right)}\left[f_{2}\left(D_{NET}\left(y_{f}\right)-D_{NET}\left(y_{r}\right)\right)\right]$$

(15) $$Loss^{\left(G_{NET}\right)}=E_{\left(y_{r},\;y_{f}\right)\sim \left(\mathbb{R},\;\mathbb{N}\right)}\left[g_{1}\left(D_{NET}\left(y_{r}\right)-D_{NET}\left(y_{f}\right)\right)\right]+E_{\left(y_{r},\;y_{f}\right)\sim \left(\mathbb{R},\;\mathbb{N}\right)}\left[g_{2}\left(D_{NET}\left(y_{f}\right)-D_{NET}\left(y_{r}\right)\right)\right]$$

From equations 14 and 15 above, we can say that *f*_1_(*D*_*NET*_ (*y*_*r*_) − *D*_*NET*_ (*y*_*f*_)) = *f*_2_ − (*D*_*NET*_ (*y*_*f*_) − *D*_*NET*_ (*y*_*r*_)). Moreover, in the case of non-saturating loss, *f*_2_(*D*_*NET*_ (*y*_*f*_) − *D*_*NET*_ (*y*_*r*_)) = *g*_1_(*D*_*NET*_ (*y*_*r*_) − *D*_*NET*_ (*y*_*f*_)), and *g*_2_(*D*_*NET*_ (*y*_*f*_) − *D*_*NET*_ (*y*_*r*_)) = *f*_1_(*D*_*NET*_ (*y*_*r*_) − *D*_*NET*_ (*y*_*f*_)). Based on the above properties, we can further simplify equations 14 and 15 as:

(16) $$Loss^{\left(D_{NET}\right)}=E_{\left(y_{r},\;y_{f}\right)\sim \left(\mathbb{R},\;\mathbb{N}\right)}\left[f_{1}\left(D_{NET}\left(y_{r}\right)-D_{NET}\left(y_{f}\right)\right)\right]$$

(17) $$Loss^{\left(G_{NET}\right)}=E_{\left(y_{r},\;y_{f}\right)\sim \left(\mathbb{R},\;\mathbb{N}\right)}\left[f_{1}\left(D_{NET}\left(y_{f}\right)-D_{NET}\left(y_{r}\right)\right)\right]$$

The generic cost functions of the relativistic average GAN for a generator and discriminator can be computed as:

(18) $$Loss^{\left(D_{NET}\right)}=E_{y_{r}\sim \mathbb{R}}\left[f_{1}\left(D_{NET}\left(y_{r}\right)-E_{y_{f}\sim \mathbb{N}}\left(D_{NET}\left(y_{f}\right)\right)\right)\right]+E_{y_{f}\sim \mathbb{N}}\left[f_{2}\left(D_{NET}\left(y_{f}\right)-E_{y_{r}\sim \mathbb{R}}\left(D_{NET}\left(y_{r}\right)\right)\right)\right]$$

(19) $$Loss^{\left(G_{NET}\right)}=E_{y_{r}\sim \mathbb{R}}\left[g_{1}\left(D_{NET}\left(y_{r}\right)-E_{y_{f}\sim \mathbb{N}}\left(D_{NET}\left(y_{f}\right)\right)\right)\right]+E_{y_{f}\sim \mathbb{N}}\left[g_{2}\left(D_{NET}\left(y_{f}\right)-E_{y_{r}\sim \mathbb{R}}\left(D_{NET}\left(y_{r}\right)\right)\right)\right]$$

In our data, TILs appear round and dark purple with deep bluish nuclei. We aimed to maintain the stain color, tissue contents, morphology, and textural details in our generated images. The relativistic average GAN cost functions did not generate output as expected. Hence, we tweaked the relativistic average GAN as follows:

(20) $$Loss_{\text{modified}}^{\left(D_{NET}\right)}=E_{y_{r}\sim \mathbb{R}}\left[\text{sum}\left\{f_{1}\left(D_{NET}\left(y_{r}\right)\right)-E_{y_{f}\sim \mathbb{N}}\left(D_{NET}\left(y_{f}\right)\right)\right\}\right]+E_{y_{f}\sim \mathbb{N}}\left[\text{sum}\left\{f_{2}\left(D_{NET}\left(y_{f}\right)\right)-E_{y_{r}\sim \mathbb{R}}\left(D_{NET}\left(y_{r}\right)\right)\right\}\right]+V\left(r,\;f\right)$$

(21) $$Loss_{\text{modified}}^{\left(D_{NET}\right)}=E_{y_{r}\sim \mathbb{R}}\left[\text{sum}\left\{g_{1}\left(D_{NET}\left(y_{r}\right)\right)-E_{y_{f}\sim \mathbb{N}}\left(D_{NET}\left(y_{f}\right)\right)\right\}\right]+E_{y_{f}\sim \mathbb{N}}\left[\text{sum}\left\{g_{2}\left(D_{NET}\left(y_{f}\right)\right)-E_{y_{r}\sim \mathbb{R}}\left(D_{NET}\left(y_{r}\right)\right)\right\}\right]$$

**FIGURE 4.**
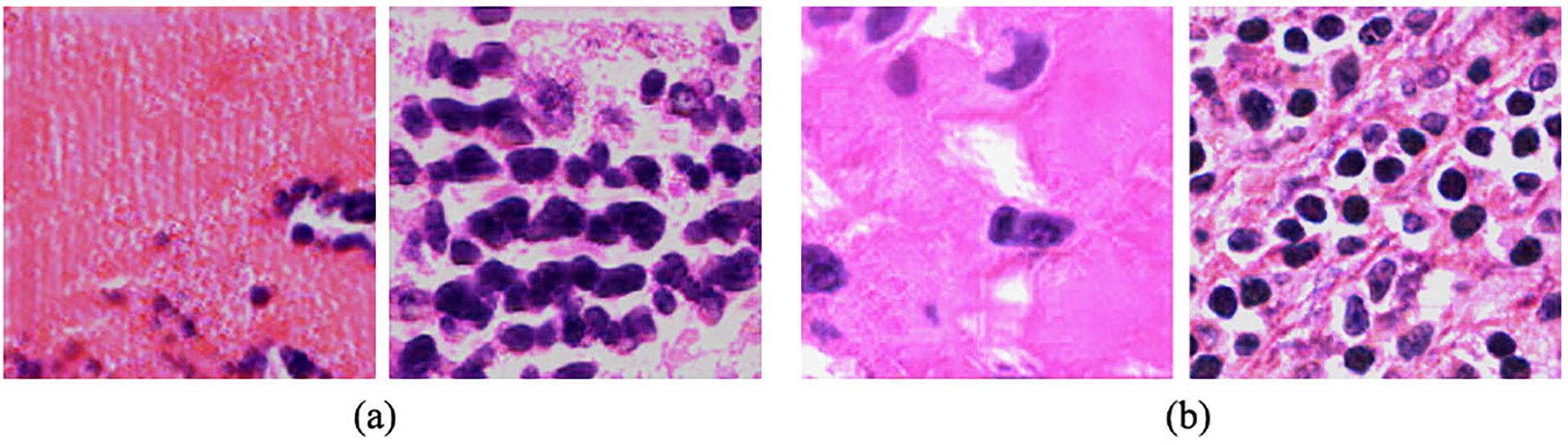
(a) Results of relativistic average GAN loss functions; (b) results of proposed loss functions.

Here, *V* (*r, f*) is computed using $\sqrt{\left\{r*\left(1-\epsilon \right)+f*\epsilon \right\}}$ where *ϵ* = *e*^−4^. Figure 4 shows the results of the relativistic average GAN loss and our proposed loss functions. The results of figure 4(b) are much smoother than the figure 4(a). Moreover, in 4(b) nuclei are easily identifiable. From the results, it is clear that our loss function generates much better result. The training and validation loss graph of *TilGAN* is shown in figure 11.

#### 3) *TilGAN* TRAINING AND TESTING PROCEDURE

The *TilGAN* architecture was trained on 267 WSIs and tested on 89 WSIs. For the training of the *TilGAN* model, we did not perform data augmentation because it would generate additional noise with poor-quality images. Hence, our suggestion is to use as many real, high-quality, hand-labeled images as the input of the generator. We set the *TilGAN* model batch size to 100 and the learning rates of *G*_*NET*_ and *D*_*NET*_ as to 1e-4 and 1e-5, respectively. The initial weights were standardized to a mean of zero with 0.02 as a standard deviation. We used the Adam optimizer with adaptive momentum. The values of *β*1 and *β*2 were set to 0.5 and 0.99, respectively. We used the TensorFlow framework for the development of *TilGAN*.

### C. CLASSIFICATION ARCHITECTURE DETAILS

Classification was performed to verify whether our synthetic images are efficient for discriminating real TIL and non-TIL patches. Our classification architecture, developed using Keras with the TensorFlow backend, was designed using six convolution layers, ReLU, two max-pooling layers, four dense layers, one flattened layer, one dropout layer, and one batch normalization layer. The details of our classification architecture are depicted in table 3.

#### 1) CLASSIFICATION MODEL TRAINING, TESTING, AND VALIDATION PROCEDURE

**FIGURE 5.**
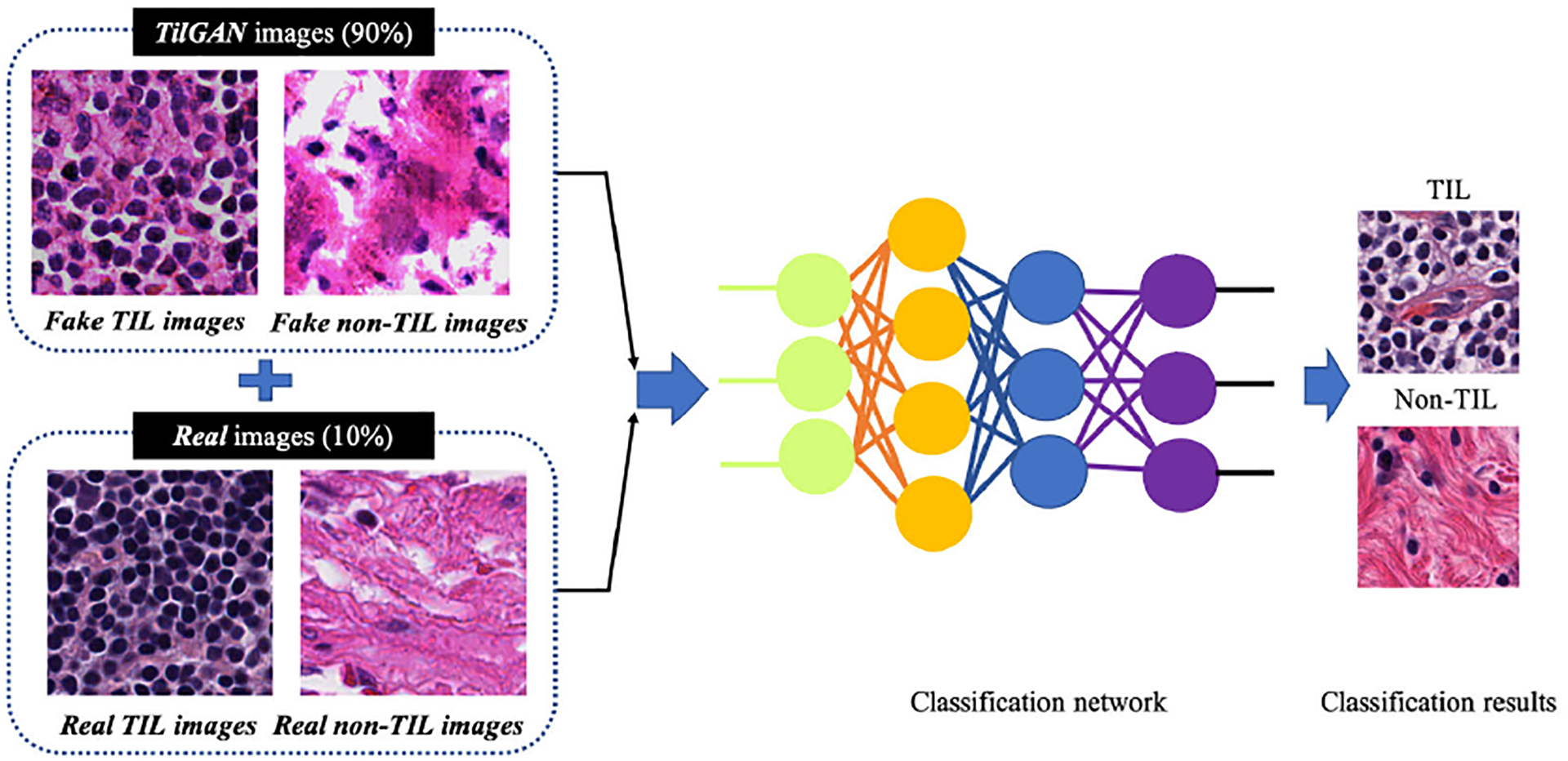
Workflow diagram of the classification model.

Figure 5 shows the classification model workflow. For the classification, we used one million high-quality synthetic image patches of size 224×224 pixels, which were generated by *TilGAN*. We added only 36 WSIs out of 356 WSIs with *TilGAN*-generated images for better classification performance. The rest of the WSIs were split into testing and validation sets to evaluate the classification model. For our classification algorithm, we used a sigmoid classifier and an Adam optimizer. The training parameters were as follows: learning rate as 0.0001, epoch as 50, dropout ratio as 0.5, and loss function as binary cross-entropy. We used rectified linear unit after each convolution layer.

### D. EVALUATION METRICS

#### 1) EVALUATION METRICS FOR *TilGAN*-GENERATED IMAGES

For the quantitative evaluation of the images generated by our proposed *TilGAN* model, we used the Inception score (IS) [15], kernel Inception distance (KID) [62], and Fréchet Inception distance (FID) [63]. All the scores were calculated using a pretrained Inception-v3 network [59], [64]. We calculated the IS as follows [59], [65]:

(22) $$\left.IS=\text{exp}\left(E_{y\sim p_{\text{image}}}KL\left(p\left(x|y\right)\|p\left(x\right)\right)\right)\right)$$

The marginal class distribution can be evaluated as:

(23) $$p\left(x\right)=\int_{y}p\left(x|y\right)p_{\text{image}}\left(y\right)$$

Here, *y* ~ *p*_*image*_ means that *y* is an image set of *p*_*image*_. *p*(*x*∣*y*) represents the conditional class distribution. KL means KL divergence.

#### 2) EVALUATION METRICS FOR THE CLASSIFICATION MODEL

The classification performances have been measured by classification accuracy, precision, recall, and F1-score [8], [66], [67]. We also computed the confusion matrix and area under the receiver operating characteristic curve.

## IV. RESULTS AND DISCUSSION

### A. RESULTS OF TilGAN

**FIGURE 6.**
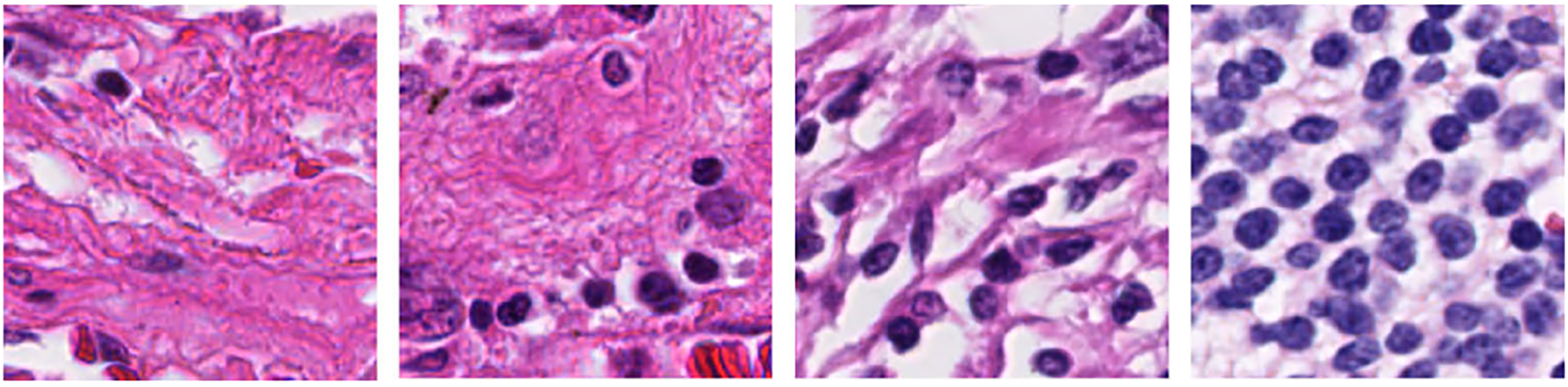
Real images of size 224 × 224 pixels.

**FIGURE 7.**
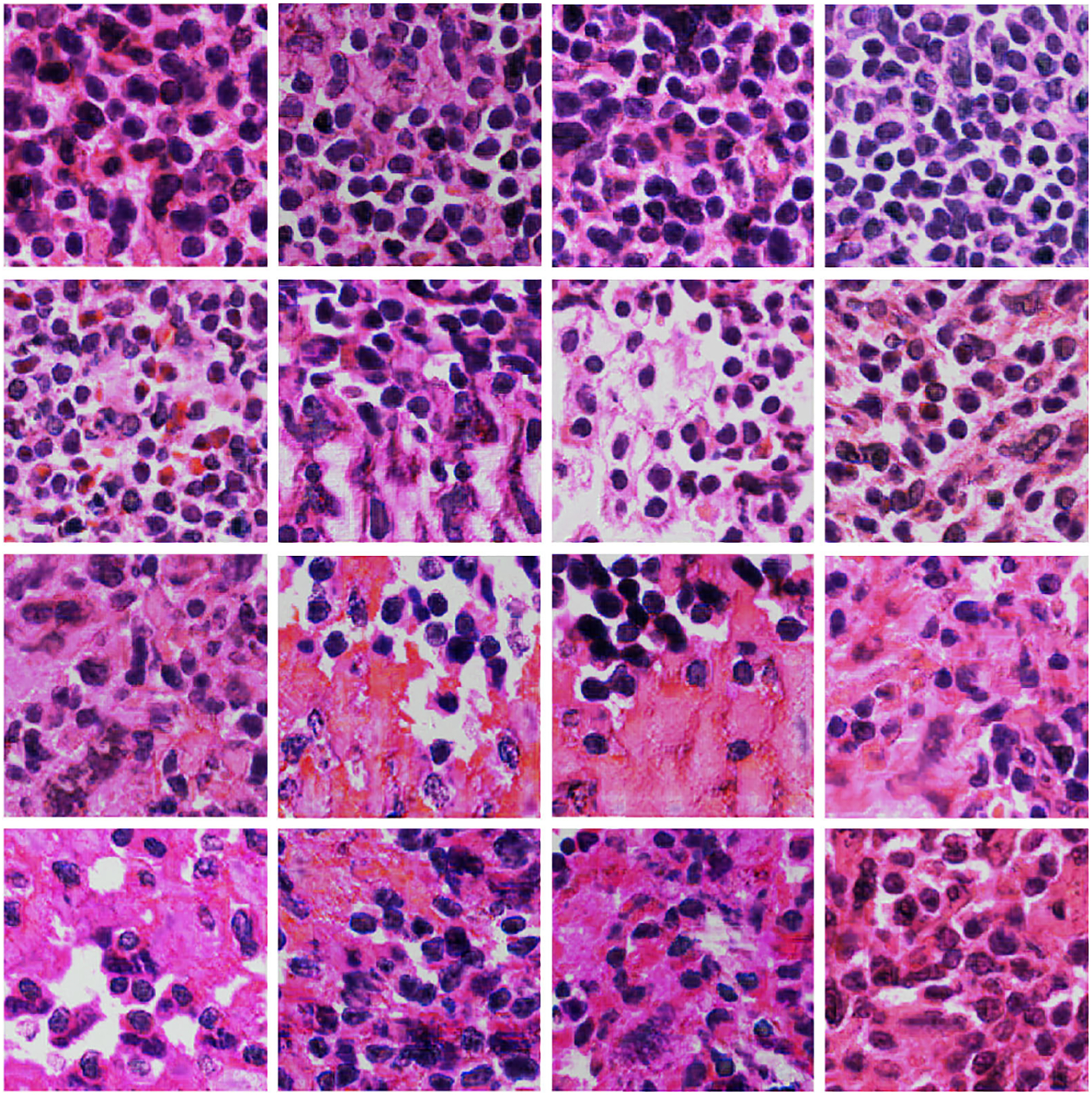
*TilGAN*-generated images of size 224 × 224 pixels.

We evaluated the quality of our proposed *TilGAN*-generated fake images through a clinical evaluation by our experts. They independently classified each image as real or fake from sets of almost 1000 images. A subset of all the real and *TilGAN*-generated fake images are shown in figures 6 and 7, respectively. Over 96% of the *TilGAN*-generated fake images were classified as real images, and all the real images were classified as real. Less than 4% of the *TilGAN*-generated fake images were classified as fake. From this experiment, it is obvious that even for an expert, it is difficult to distinguish *TilGAN*-generated fake images from a mixture of fake and real data. This finding means that the *TilGAN* architecture generates high-quality images and maintains the proper stain color with a significant amount of tissue content based on the tumor stage.

Moreover, we also evaluated the quality and diversity of the *TilGAN*-generated fake images by the most popular quantitative evaluation metrics for GANs, i.e., the Inception score, Fréchet Inception distance, and kernel Inception distance. The Inception score was used to evaluate the quality and diversity of the fake images. A high Inception score indicates that the generated fake image contains high-density and clear objects for all classes. However, this scoring technique has a few disadvantages. One of the main disadvantages is that it does not use the statistics of real data. To overcome this issue, we used the Fréchet Inception distance and kernel Inception distance. The Fréchet Inception distance has been used to calculate the distance between Inception feature vectors for fake and real images. The value of the Fréchet Inception distance changes with the image diversity, as it is robust to noise. If a dataset contains many diverse images, the Fréchet Inception distance will be low or closer to zero. On the other hand, if the image diversity between the real and synthetic images decreases, the Fréchet Inception distance will be high. The kernel Inception distance has been used to evaluate the similarity between real and fake images [62]. If the kernel Inception distance is low, the real and fake images are very similar to each other, or it is very hard to distinguish them from a mixture of real and fake images.

**FIGURE 8.**
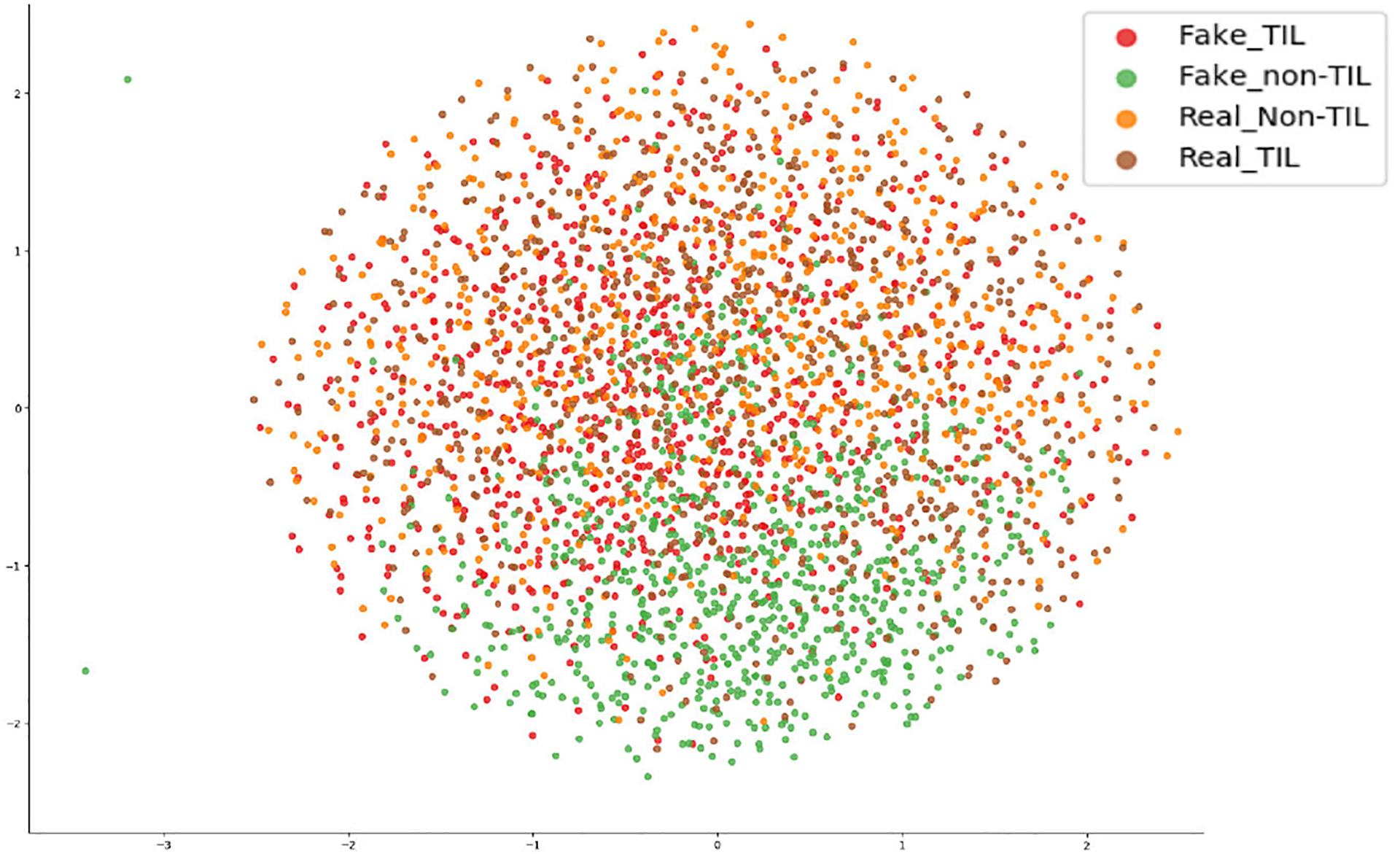
t-SNE graph.

The results of the Inception score, Fréchet Inception distance, and kernel Inception distance are shown in table 4. We calculated the Inception score on both real and *TilGAN-*generated fake images because it only uses one kind of image at a time. We achieved an Inception score of 2.32±0.02 (mean ± standard deviation) for the real images and an Inception score of 2.90±0.04 (mean ± standard deviation) for the fake images. This finding indicates that the *TilGAN*-generated fake images contain high-density tissues and clear objects and or more diverse. For the Fréchet Inception distance and kernel Inception distance measurements, we used the outputs of the last hidden layer, i.e., the pooling layer, of the same pretrained Inception-v3 network [64]. The Fréchet Inception distance is 0.312, which is very close to zero, and the kernel Inception distance is 1.44±0.025. These two values are lower than the Inception scores of the real and fake data. Undoubtedly, the *TilGAN* generates a more diverse and high-quality dataset, which is almost similar to the real images. The real and fake data distribution is shown using the t-stochastic neighbor embedding (t-SNE) plot in figure 8. This plot gives a good understanding of the visual and color similarities of the generated synthetic images with the real images. The TilGAN generates wide varieties of fake non-TIL patches, which also include some white patches. Hence, some green dots are away from the dense population.

### B. TIL AND NON-TIL IMAGE CLASSIFICATION RESULTS

**FIGURE 9.**
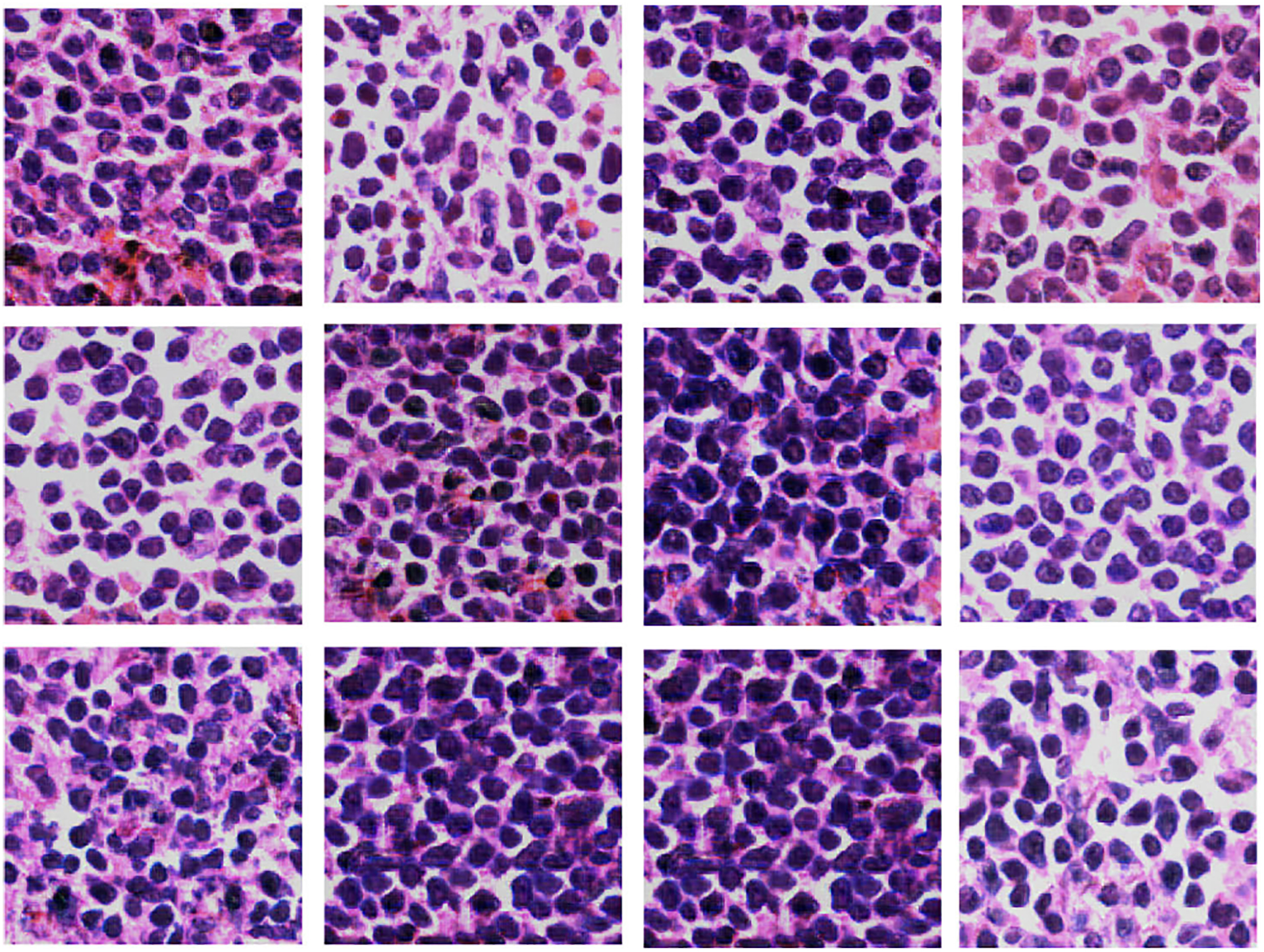
*TilGAN*-generated tumor-infiltrating lymphocyte images of size 224 × 224.

**FIGURE 10.**
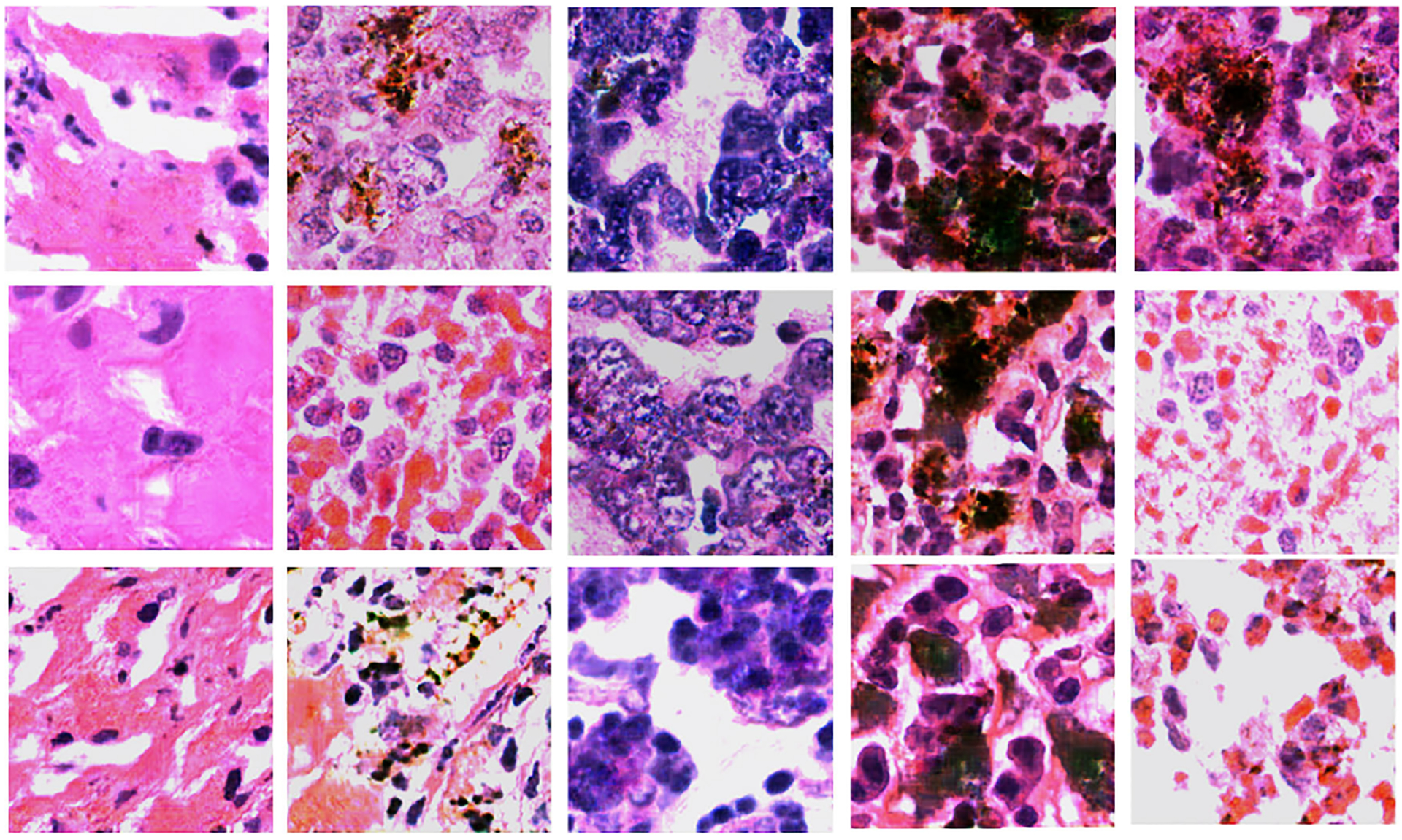
*TilGAN*-generated non-tumor-infiltrating lymphocyte images of size 224 × 224.

**FIGURE 11.**
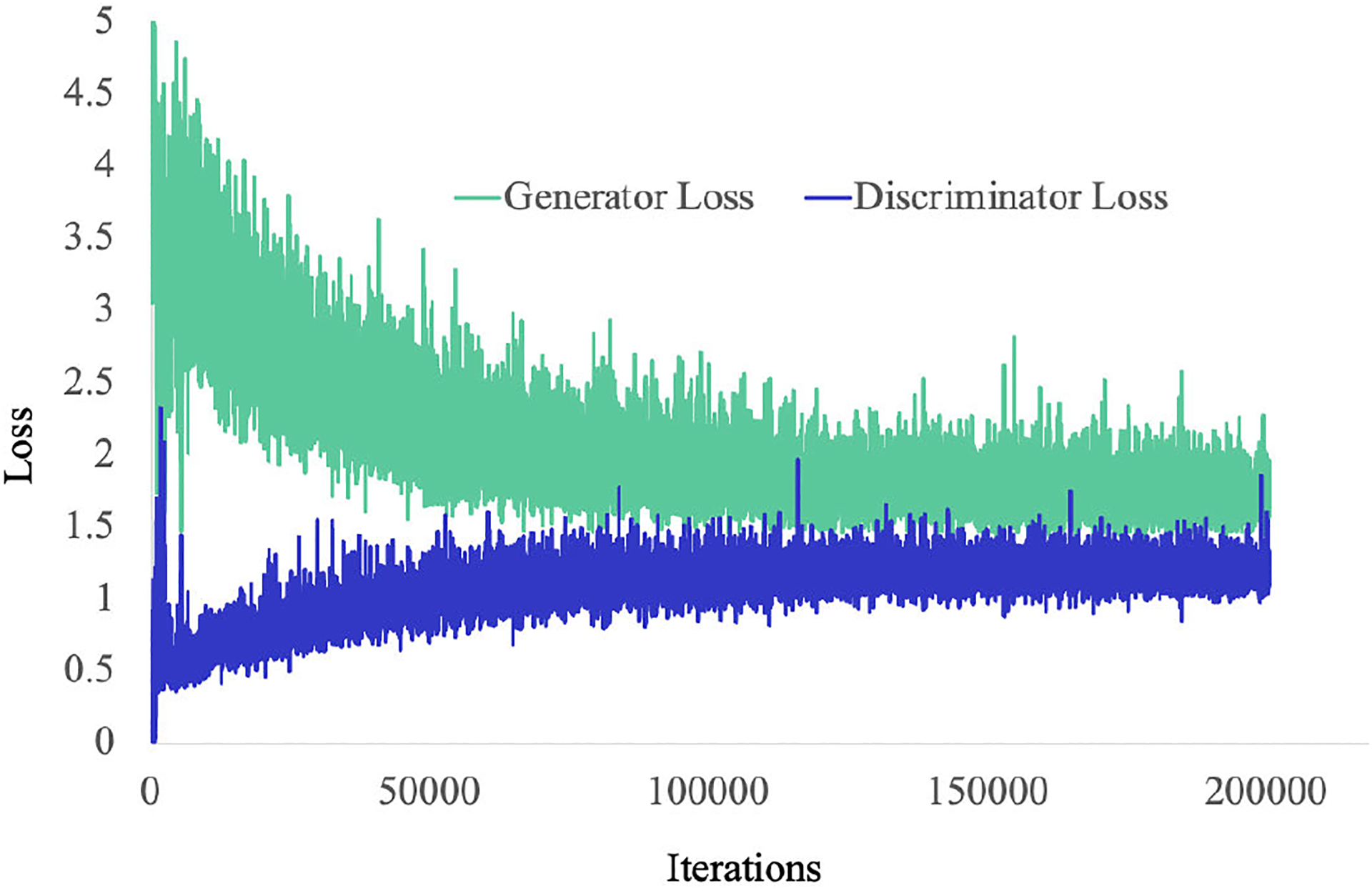
Training and validation loss for the *TilGAN* model.

**FIGURE 12.**
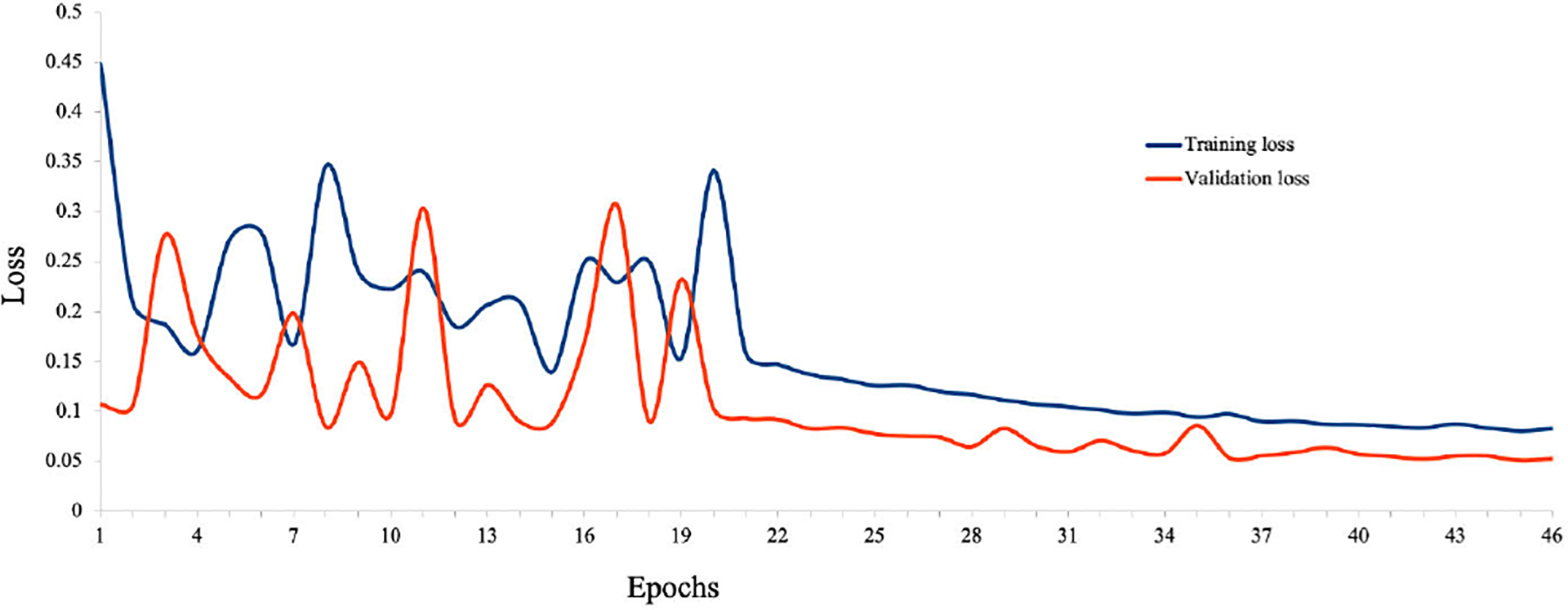
Training and validation loss for the TIL and non-TIL classification model.

In the previous sections, we have shown the results of the qualitative and quantitative analysis of *TilGAN*. In this section, we will show the performance of our classification model, where 90% (i.e., one million) *TilGAN*-generated images and 10% (i.e., 36 WSIs) real hand-labeled images were used for model training for distinguishing real TIL and non-TIL patches. Testing and validation of our trained model was performed using only real images with zero overlap. The classification model was run for up to 50 epochs, and the model started converging after 41 epochs. The training and validation losses of our classification model are shown in figure 12. A subset of the *TilGAN*-generated synthetic TIL images and non-TIL images are shown in figures 9 and 10, respectively. Figure 15 shows an accuracy plot of the real, fake, and combined outcomes. In this plot, when only fake images were used, we observed the unstable behavior of the model’s performance from epochs 16 to 21. However, this behavior is normal for any classification model. This finding indicates that our proposed model is learning, and based on the quality of the batch images, the learning performance varies. We also noticed that after 36 epochs, the accuracy plots of the real, fake, and combined outcomes converged. However, their accuracy levels are different.

The accuracy for the real images is comparatively lower than that of the proposed model (where 90% fake and 10% real images were used). The main reason for this behavior is that we only used a minimal number of real hand-labeled data (i.e., 10%) for model training. Significant changes in the accuracies were not observed when only fake and combined (90% fake and 10% real) images were used for model training.

The training scheme was repeated ten times with different data as per the Monte Carlo cross-validation criteria. Each time, the training and testing dataset was split randomly, but the same principle applies. We achieved an average classification accuracy of 97.83%, an F1-score of 97.37%, a precision of 98.34%, and a recall of 96.49%. Table 5 shows the 10-fold cross-validation results. We also computed the confusion matrix on 18,400 image patches (8750 TIL and 9650 non-TIL patches) of size 224 × 224 pixels. The confusion matrix is shown in figure 16. Figure 17 shows a receiver operating characteristic curve, which has an area under the curve of 97%. From the above results, we can say that our classification model accurately classifies the real TIL and non-TIL patches.

**FIGURE 13.**
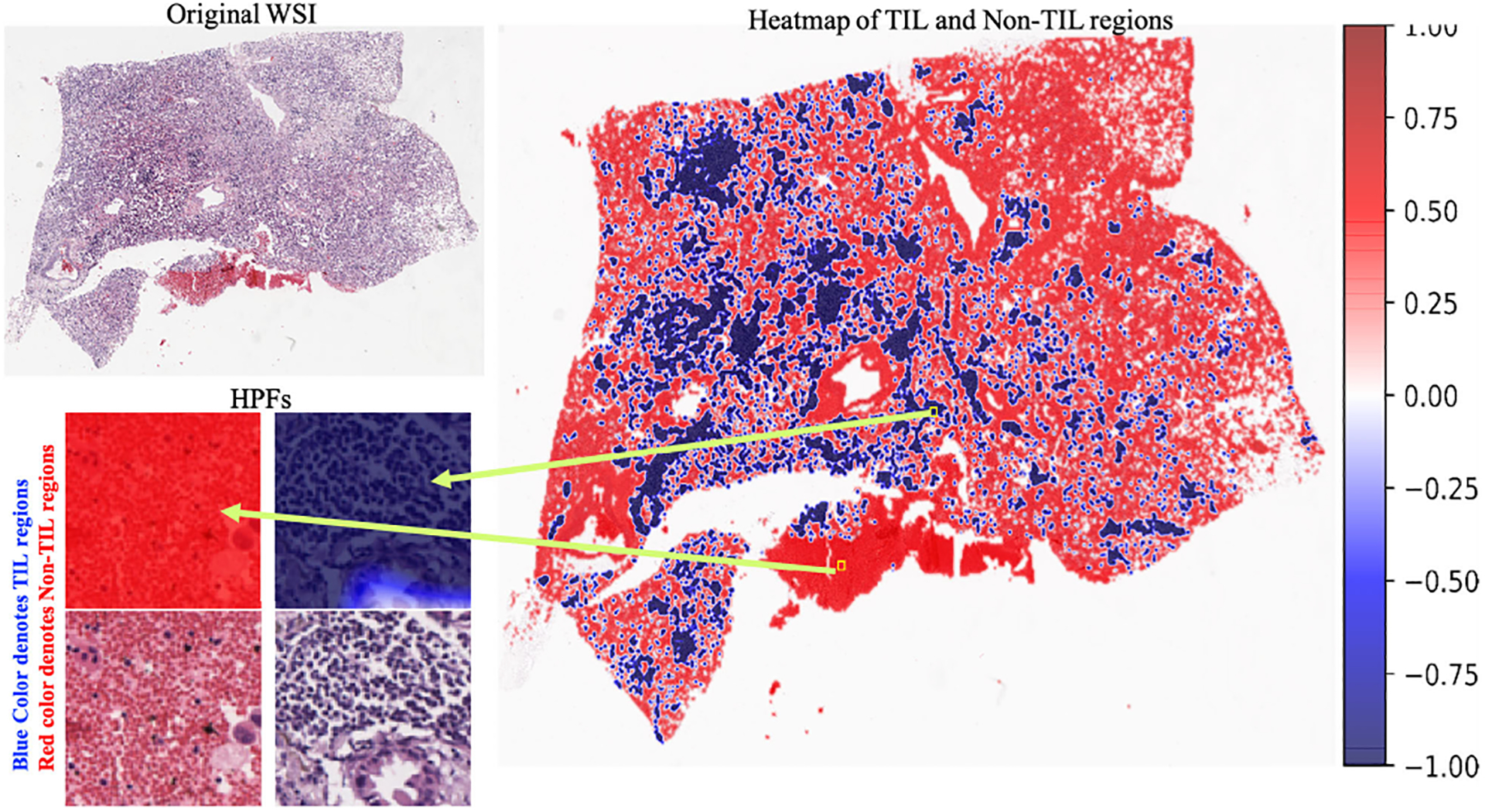
TIL classification results on a single whole-slide image.

**FIGURE 14.**
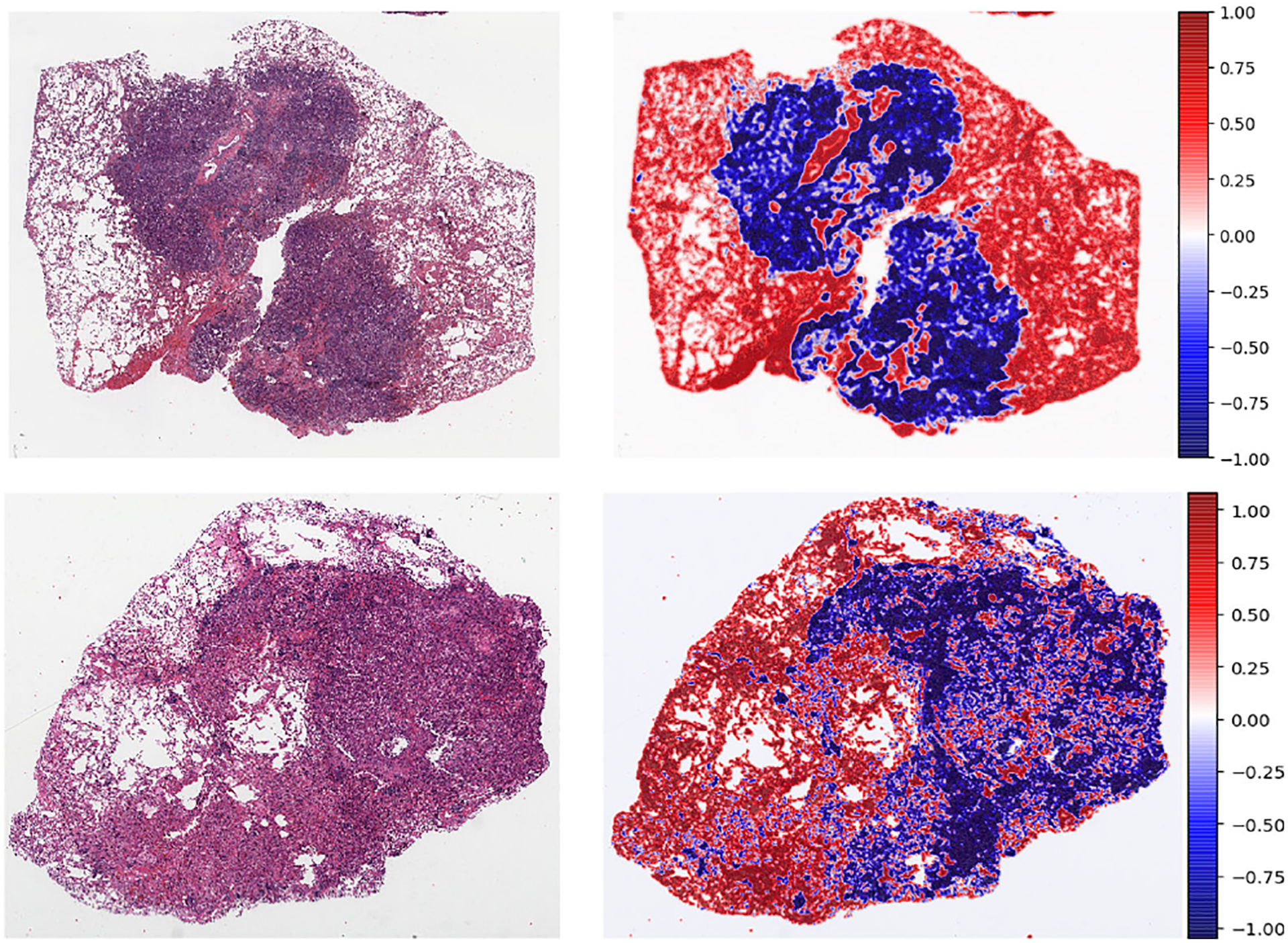
TIL classification results.

**FIGURE 15.**
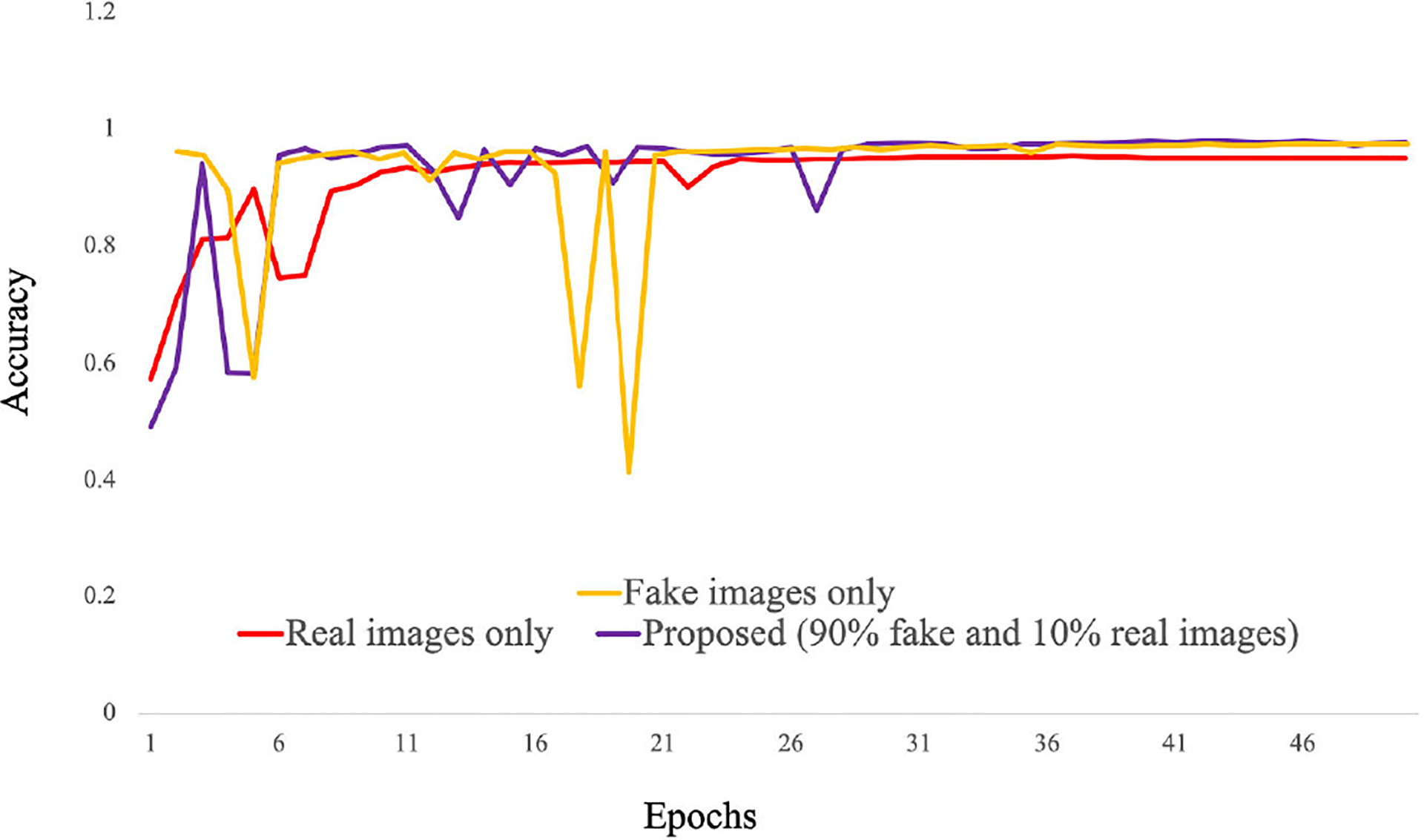
Accuracy curves.

**FIGURE 16.**
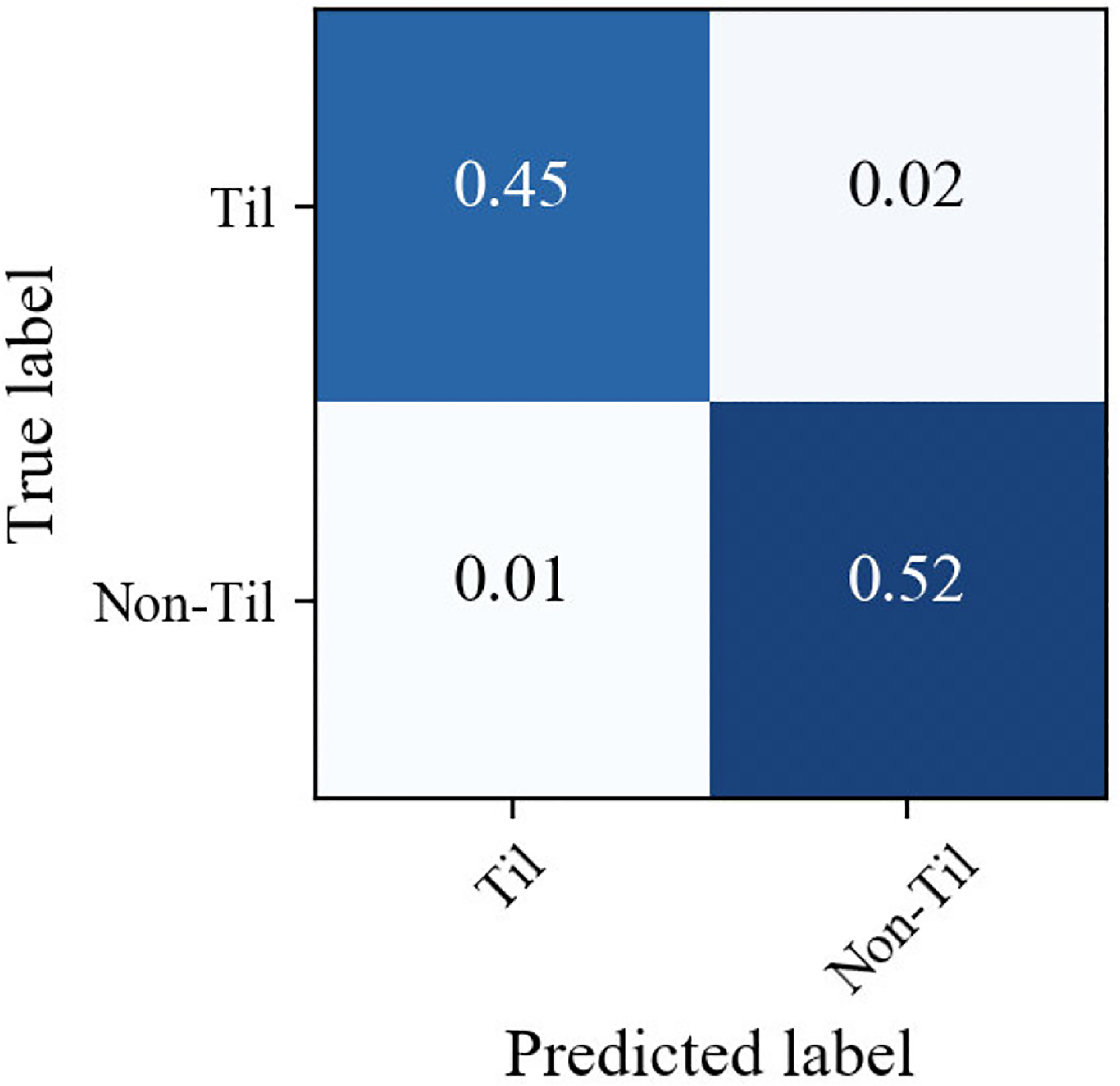
Confusion matrix between the predicted label and true label.

**FIGURE 17.**
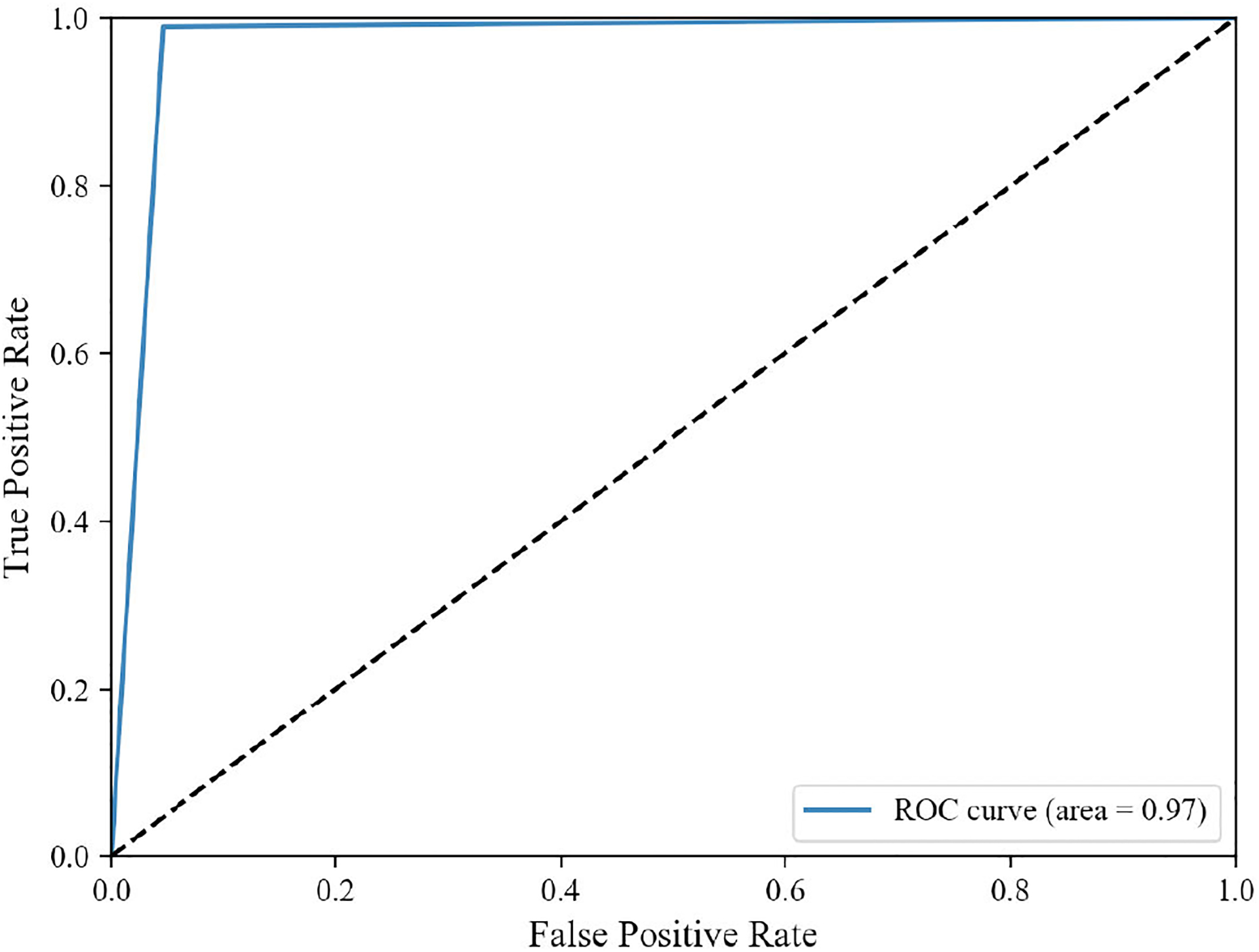
Receiver operating characteristic curve.

The classification results on the whole-slide pathology images are shown in figures 13 and 14 using a heat map. For heat map generation, the first whole-slide images were tiled into 224 × 224 pixels. Next, the probability score was calculated for each tile using our trained classification model. The probability score determines the probability of having TILs or non-TILs in a specific tile. In our experiment, 0 indicates a TIL tile, and 1 indicates a non-TIL tile. When the probability score was close to zero, then the tile was considered a TIL tile, and when the probability score was close to 1, then the tile was considered a non-TIL tile. In figure 13, red and blue regions from the heat map were separately highlighted and matched with the original WSI. The red and blue regions represent the non-TIL and TIL regions, respectively, of the original WSI. In figure 14, we show the heat map representation of the classification scores for two other whole-slide images.

## V. CONCLUSION

In this study, we proposed the *TilGAN* model for improving the quality of synthetic pathology images. Our proposed architecture differs from existing GANs mainly because of architecture and loss functions. *TilGAN* does not contain an attention layer, similar to the Pathology GAN architecture [49]. In the *TilGAN* model, the numbers of layers in the generator and discriminator are different. Because of these properties, we can maintain the quality and quantity of specific types of target objects; in our case, it is TIL in each tile. The generated TIL patches are mostly covered by lymphocytes rather than stroma or other artifacts. Similarly, non-TIL patches are mostly covered by stroma and other artifacts rather than lymphocytes. This phenomenon is obviously a good sign of our architecture. This finding shows that we are not using up our resources on generating low-quality images. Another interesting point is our loss functions, which maintain the features of TIL morphology, texture, and color of real images in the synthetic images. Hence, image normalization, enrichment, and translation are not essential as in the approach proposed by [46]. We properly verified the quality of our synthetic images by the Inception score, kernel Inception distance, and Fréchet Inception distance metrics, which showed promising results. We plotted the t-SNE graph using real and synthetic images, which showed a strong correlation between the real and synthetic images. Next, the generated synthetic images were physically verified by experts. They faced difficulties in distinguishing the real and synthetic images from the mixture of real and synthetic images. The use of one million synthetic images for training the classification model was an additional evaluation measure for the *TilGAN* model. Here, we showed that the *TilGAN*-generated images can efficiently classify real TIL and non-TIL patches with improved accuracy. From the various image verification methods, we proved the usefulness and effectiveness of our proposed *TilGAN* architecture. Therefore, we can say that our approach performs better in generating TIL and non-TIL images than other methods. In the future, this architecture can be used to generate radiology and other non-clinical data.

## Figures and Tables

**TABLE 1.** Characterization of existing GAN architectures.

Purpose	*GAN architectures*
Image synthesis	DCGAN [17], [18]; LostGAN [19]; StackGAN [20]; Standard GAN [21]; Cycle-consistency GAN [22]; Conditional GAN [23]
Image translation	MedGAN [24]; Cycle-consistency GAN [25]; Conditional GAN [26]
Image conversion	Context-aware GAN [27]; Cycle-consistency GAN [28]
Image enhancement and color normalization	Conditional GAN [29]; Cycle-consistency GAN [30]; Conditional GAN [31]
Style transferring	StyleGAN [32]; Conditional GAN [33]

**TABLE 2.** Proposed *TilGAN* architecture.

TilGAN Generator Network						
No. of layers	Layer type	Channels	Filter Size	Stride	Padding	Output Shape
14	Generated Image	3	3	1	SAME	(?,224,224,3)
13	Convolution	32	2	1		(?,224,224,32)
12	Up-Convolution	32	1	2		(?,224,224,32)
11	Convolution	64	2	1		(?112,112,64)
10	Up-Convolution	64	1	2		(?,112,112,64)
9	Convolution	128	2	1		(?,56,56,128)
8	Up-Convolution	128	1	2		(?,56,56,128)
7	Convolution	256	2	1		(?,28,28,256)
6	Up-Convolution	256	1	2		(?,28,28,256)
5	Convolution	512	2	1		(?,14,14,512)
4	Up-Convolution	512	1	2		(?,14,14,512)
3	Convolution	256	2	1		(?,7,7,256)
2	Dens Layer	12544	-	-	-	(?,12544)
1	Dens Layer	1024	-	-	-	(?,1024)
TilGAN Discriminator Network						
No. of layers	Layer type	Channels	Filter Size	Stride	Padding	Output Shape
1	Convolution	3	2	1	SAME	(?,224,224,3)
2	Down-Convolution	16	4	2		(?,112,112,16)
3	Convolution	16	2	1		(?,112,112,16)
4	Down-Convolution	32	4	2		(?,56,56,32)
5	Convolution	32	2	1		(?,56,56,32)
6	Down-Convolution	64	4	2		(?,28,28,64)
7	Convolution	64	2	1		(?,28,28,64)
8	Down-Convolution	128	4	2		(?,14,14,128)
9	Convolution	128	2	1		(?,14,14,128)
10	Down-Convolution	256	4	2		(?,7,7,256)
11	Convolution	256	2	1		(?,7,7,256)
12	Dow n-Convolution	512	4	2		(?,4,4,512)
13	Dens Layer	1024	-	-	-	(?,1024)
14	Dens Layer	1	-	-	-	(?,1)

**TABLE 3.** Classification architecture.

No. of layers	Layer type	Channels	Filter Size	Output Shape
1	Input	3	-	(?,224,224,3)
2	Convolution	180	3	(?,222,222,180)
3	Convolution	160	3	(?,220,220,160)
4	Max-pooling	160	5	(?,44,44,160)
5	Convolution	160	3	(?,42,42,160)
6	Convolution	140	3	(?,40,40,140)
7	Convolution	100	3	(?,38,38,100)
8	Convolution	50	3	(?,36,36,50)
9	Max-pooling	50	5	(?,7,7,50)
10	Flatten	-	-	(?,2450)
11	Dense	-	-	(?,180)
12	Dense	-	-	(?,100)
13	Dense	-	-	(?,50)
14	Dropout	-	-	(?,50)
15	Batch Normalization	-	-	(?,50)
16	Dense	-	-	(?,1)

**TABLE 4.** Performance comparison between pathology GAN and the proposed method (*TilGAN*) using evaluation metrics (IS, KID, and FID).

Types of data	*IS*	*KID*	*FID*
*Real Data*	2.32±0.02	-	-
Pathology GAN [49]	2.75±0.012	4.66±0.48	1.07±0.023
***TilGAN***	***2.90±0.04***	***1.44±0.025***	***0.312±0.001***

**TABLE 5.** Ten-fold cross-validation results.

Folds	Accuracy	F1-score	Precision	Recall
Fold-1	0.98	0.98	0.98	0.98
Fold-2	0.98	0.98	0.98	0.97
Fold-3	0.98	0.98	0.99	0.96
Fold-4	0.97	0.97	0.97	0.97
Fold-5	0.98	0.97	0.99	0.96
Fold-6	0.98	0.98	0.98	0.98
Fold-7	0.97	0.96	0.99	0.93
Fold-8	0.98	0.97	0.99	0.96
Fold-9	0.97	0.97	0.97	0.96
Fold-10	0.98	0.97	0.98	0.97
***Average***	***0.98***	***0.97***	***0.98***	***0.96***
